# Virome Diversity among Mosquito Populations in a Sub-Urban Region of Marseille, France

**DOI:** 10.3390/v13050768

**Published:** 2021-04-27

**Authors:** Amira Nebbak, Sonia Monteil-Bouchard, Jean-Michel Berenger, Lionel Almeras, Philippe Parola, Christelle Desnues

**Affiliations:** 1 IHU-Méditerranée Infection, 13005 Marseille, France; amiranebbak@yahoo.fr (A.N.); jmberenger@free.fr (J.-M.B.); almeras.lionel@gmail.com (L.A.); philippe.parola@univ-amu.fr (P.P.); 2 Aix Marseille Université, Intitut de Recherche pour le Développement (IRD), Assistance Publique-Hopitaux de Marseille (AP-HM), Service de Santé des Armées (SSA), Vecteurs Infections Tropicales et Méditerranéennes (VITROME), 13005 Marseille, France; 3 Centre de Recherche Scientifique et Technique en Analyses Physico-Chimiques (CRAPC), BP 384, Zone Industrielle, Bou-Ismail RP 42004, Tipaza, Algeria; 4 Aix Marseille Université, Intitut de Recherche pour le Développement (IRD), Assistance Publique-Hopitaux de Marseille (AP-HM), Microbes Evolution Phylogeny and Infections (MEPHI) UM 63, 13005 Marseille, France; sonia.bouchard@mio.osupytheas.fr; 5 Aix-Marseille Université, Université de Toulon, Centre National pour la Recherche Scientifique (CNRS), Intitut de Recherche pour le Développement (IRD), Mediterranean Institute of Oceanography (MIO) UM 110, 13288 Marseille, France; 6 Unité de Parasitologie et Entomologie, Département des Maladies Infectieuses, Institut de Recherche Biomédicale des Armées, 13005 Marseille, France

**Keywords:** mosquitoes, virome, metagenomics, diversity, France

## Abstract

Some mosquito species have significant public health importance given their ability to transmit major diseases to humans and animals, making them the deadliest animals in the world. Among these, the *Aedes* (*Ae*.) genus is a vector of several viruses such as Dengue, Chikungunya, and Zika viruses that can cause serious pathologies in humans. Since 2004, *Ae. albopictus* has been encountered in the South of France, and autochthonous cases of Dengue, Chikungunya, and Zika diseases have recently been reported, further highlighting the need for a comprehensive survey of the mosquitoes and their associated viruses in this area. Using high throughput sequencing (HTS) techniques, we report an analysis of the DNA and RNA viral communities of three mosquito species *Ae. albopictus*, *Culex* (*Cx*.) *pipiens*, and *Culiseta* (*Cs*.) *longiareolata* vectors of human infectious diseases in a small sub-urban city in the South of France. Results revealed the presence of a significant diversity of viruses known to infect bacteria, plants, insects, and mammals. Several novel viruses were detected, including novel members of the *Rhabdoviridae*, *Totiviridae*, *Iflaviviridae*, *Circoviridae*, and *Sobemoviridae* families. No sequence related to major zoonotic viruses transmitted by mosquitoes was detected. The use of HTS on arthropod vector populations is a promising strategy for monitoring the emergence and circulation of zoonoses and epizooties. This study is a contribution to the knowledge of the mosquito microbiome.

## 1. Introduction

Mosquitoes (Diptera: *Culicidae*) are an insect family comprising more than 3600 species [1]. Many species are major vectors of infectious agents to humans and animals, like parasites [2], viruses [3], and potentially bacteria [4,5]. Mosquito-borne viruses have been estimated to cause over 100 million cases of human disease annually [6]. These last decades, several emerging viruses transmitted by mosquitoes have been spreading more widely and affecting larger populations worldwide [7].

The south of France is now known as a potential risk area for mosquito-borne viral infections. After its introduction in the French Riviera in 2004, the Asian tiger mosquito *Ae. albopictus* [8,9] has propagated into the country. It is particularly endemic in the South of France. There, several autochthonous cases of Dengue and Chikungunya have been identified following the introduction of the viruses with returning sick travelers and transmission to other people by local *Ae. albopictus* [10,11,12,13]. During the summer of 2018, 20 human cases of West Nile virus (WNV) infections were also reported in the PACA region. WNV is a neurotropic virus vectored by mosquitoes of the *Culex* genus to amplifying host (birds) and, accidentally, to dead-end hosts such as humans and horses [14]. Very recently, in October 2019, two cases of locally acquired Zika virus (ZIKV) disease in the Var department were laboratory-confirmed [15,16]. These are the first cases of autochthonous, vector-borne transmission of ZIKV in Europe; investigations are ongoing to find other possible cases and to avoid further transmission [15,16].

In recent years, HTS and the “vector enabled metagenomics approach” have emerged as promising tools for virus surveillance using arthropods as a template. Viral metagenomics has proved to be an interesting tool for zoonotic disease control [17,18]. Several studies have been conducted on arthropods collected around the world, including mosquitoes [19,20,21,22], ticks [23], biting midges [24], and other invertebrates [25], enabling the discovery of several new viruses belonging to different viral families, for which the potential risk to humans remains mostly unknown [26]. A very recent study demonstrated that a single mosquito could be used for mosquito virome exploration [22]. Some discovered viruses are considered insect-specific viruses (ISVs) and seem to play a role in regulating the transmission of pathogenic arboviruses in coinfected mosquitoes [27]. Their possible potential application as biocontrol and vaccine agents against mosquito-borne classical arboviruses have been evoked [26]. On the other hand, the success of vector control and epidemiological vector surveillance strategies are conditioned by correct and precise identification of the vectors. Recently, MALDI-TOF MS was relevantly applied for arthropods identification, including mosquitoes [28]. This innovative proteomic tool is now recognized to be compatible with field investigation [29,30].

In this context, the present study explores the DNA and RNA viromes of mosquito populations in a sub-urban area of the large Marseille city, South of France. Mosquitoes were first submitted to MALDI-TOF MS for precise identification and classification prior to performing viral metagenomics analysis.

## 2. Materials and Methods

### 2.1. Study Area and Mosquito Collection

**Figure 1:**
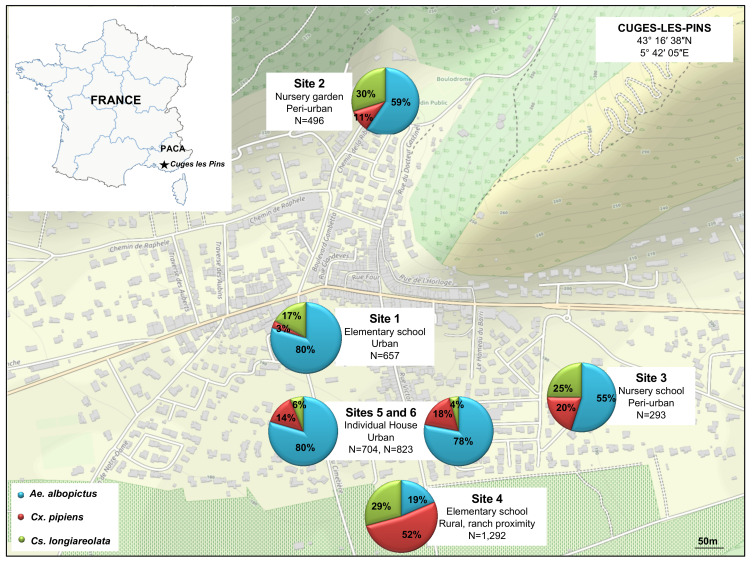
Mosquito collection sites. Map of Cuges-les-Pins area (France) indicated the location of the six sampling sites and the proportion of each of the three mosquito species collected. *Ae. albopictus* (**blue**), *Cx. pipiens* (**red**), and *Cs*. *longiareolata* (**green**).

This study was conducted from April to November 2016 in the village of Cuges-les-Pins (5000 habitants), in the south of France, located 30 km east of Marseille. This area was chosen based on complaints of mosquito nuisances provided by the citizens and registered by the Marseille city healthcare department and its large horse population (>5000 horses). Mosquitos were sampled using BG-sentinel traps (Biogents AG, Weissenburgstr, Regensburg, Germany), placed in six sampling sites (Figure 1). Once a week, each trap bag was retrieved and replaced by another. Details about the sites and mosquito collection are available in Figure 1.

### 2.2. Morphological Mosquito Identification

Mosquitos were separated from the other arthropod families collected in the trap. They were sorted per genus, sex and were counted prior to perform morphological identification under Binocular loupe (Leica microsystems, Wetzlar, Germany), using dichotomous keys [31]. All samples were rinsed with alcohol and 0.02 µm filtered water to remove environmental contaminants and stored in 1.5 mL Eppendorf tubes at −80 °C until processing. For each mosquito specimen, the species, gender, engorgement status, trapping date, and location were recorded in a spreadsheet. The experimental design is detailed in Supplementary File S1.

### 2.3. MALDI-TOF MS

Each week, the legs from six specimens morphologically identified per species and per collection site were submitted to MALDI-TOF MS analysis for identification, as previously reported [32]. Protein mass profiles were obtained using a Microflex LT MALDI-TOF Mass Spectrometer (Bruker Daltonics, Germany). The Resulting MS profiles were visualized with Flex analysis v.3.3 software and exported to ClinProTools version v.2.2 and MALDI-Biotyper v.3.0 (Bruker Daltonics, Germany) for data processing (smoothing, baseline subtraction, peak picking). The MS spectra from selected adult mosquitoes were tested against the homemade MS reference spectra database, including, among other arthropods reference spectra, 201 MS reference spectra from 31 distinct adult mosquito species [33]. The reliability of species identification was estimated using the log score values (LSVs) obtained from the MALDI Biotyper software v.3.3, which ranged from 0 to 3. These LSVs corresponded to the degree of similarity between spectra being tested and the MS reference spectra database. A specimen was considered to be correctly and relevantly identified at the species level when the queried spectrum reached the threshold log score value (LSV) of 1.8 [28].

### 2.4. Virome Preparation

Mosquitoes were grouped in three pools for virome analyses: pool A (100 non-engorged female *Ae. albopictus*); pool B (100 non-engorged female *Cx. pipiens*), and pool C (100 non-engorged female *Cs. longiareolata*). For each pool, mosquitoes collected at distinct time points per site were selected (Table 1). The composition of each pool is detailed in Supplementary File S2. Uniquely visually non-engorged female mosquitoes were used in this study to avoid viral sources from recent blood meals. The three mosquito pools were crushed with two 3 mm tungsten beads using a tissue lyser device at 25 Hz for two minutes (Qiagen, Courtaboeuf, France). The clarified supernatant was subsequently used as a template for virome preparation, as previously described [34]. Briefly, the clarified supernatant was filtered through a 0.45 µm filter (Millipore, Molsheim, France), and free nucleic acids were digested with a cocktail of nucleases. Finally, the digested supernatant was purified onto a discontinuous 30%–66% sucrose gradient and ultracentrifuged at 32,000 rpm for 4 h at 4 °C on a Sw32Ti rotor (Beckman-Coulter, Villepinte, France). The viral fraction was harvested at the interface between the 30% and 66% sucrose layers. Total viral nucleic acids were extracted from the purified viral fraction with a high pure viral nucleic acid large volume kit (Roche Applied Science, Mannheim, Germany), according to the manufacturer’s recommendations. Random amplification was performed using the Froussard random RT-PCR [35] and a Genomiphi V3 kit (GE Healthcare, Vélizy-Villacoublay, France) in two independent reactions [36], for viral RNA and DNA, respectively. Amplification products were purified with Agencourt AMPure beads (Beckman-Coulter, Villepinte, France) according to the manufacturer’s protocol, eluted to a final volume of 15 µL and sequenced using MiSeq Technology using paired-end and barcode strategies according to a Nextera XT library kit in a 2 × 250 bp format (Illumina Inc., San Diego, CA, USA).

### 2.5. Transmission Electron Microscopy (TEM)

The viral fractions of the pools A, B, and C mosquito samples were concentrated for TEM. The viral fraction was supplemented with PBS and loaded sucrose fraction on Amicon Ultra 4 mL Centrifugal filters (Merck Millipore Ltd., Cork, Ireland). The viral fraction was then centrifuged 2 min at 3000× *g*, rinsed with PBS and centrifuged again until obtaining approximately 50 µL of viral suspension, put for 1 min into liquid nitrogen and quickly at −80 °C, then fixed for one hour at 4 °C with 2.5% glutaraldehyde. The fixed viral fraction was then diluted to a final volume of 4 mL in PBS and directly adsorbed onto formvar carbon films on 400 mesh nickel grids (FCF400-Ni, EMS) by ultracentrifugation at 130,000 g for one hour at 4 °C, as previously described [37]. Grids were stained for 10 s with 1% molybdate solution in filtered water at room temperature. Electron micrographs were obtained on a Tecnai G2 transmission electron microscope (FEI) operated at 200 keV equipped with a 4096 × 4096 pixel resolution Eagle camera (FEI).

### 2.6. Bioinformatic Analyses of the Viromes

Raw reads were imported into the CLC Genomics Workbench 6.0.1 program (CLC Bio, Aarhus, Denmark) and trimmed according to their quality score, the presence of ambiguities, and their length (reads shorter than 50 nt were discarded). Trimmed reads were compared to the NCBI non-redundant (nr) protein database using the BlastX algorithm using DIAMOND software [38]. The results were visualized using MEGAN software [39] to see the relative abundances of each sequence present in a given sample. Trimmed paired-reads were then assembled into contigs using the CLC Genomics program following the default settings. Contigs were compared to the NCBI non-redundant protein database using the BlastX algorithm with DIAMOND software [38]. The results were observed using the MEGAN software [39]. The open reading frames (ORFs) present in these contigs were determined by MetaGeneMark according to the default heuristic parameters [40]. Translated ORFs were compared to the NCBI nr database by BlastP using the DIAMOND software, and the results were observed with MEGAN (Supplementary File S1).

### 2.7. Phylogenetic Analyses

The open reading frames (ORFs) were aligned with other amino-acid sequences retrieved from the GenBank database using MUSCLE aligner (v3.8.31) [41] implemented through MEGA7 configured for the highest accuracy (MUSCLE with default settings) [42]. The amino-acid substitutions models that best fitted the data were performed using the Model test implemented in the software MEGA7 and were considered for all phylogenetic analyses. The phylogenetic trees were reconstructed using the maximum likelihood method implemented in the PhyML program (v3.1/3.0). Graphical representation and edition of the phylogenetic tree were performed with TreeDyn (v198.3).

### 2.8. Isolation on Vero and C6/36 Cells

Virus isolation was carried out using Vero cells (African green monkey kidney cells) and C6/36 (*Ae. albopictus*) using the viral sucrose fraction of the pool A containing *Ae. albopictus*. The cultures were maintained at 28 °C and 37 °C for C6/36 and Vero cells, respectively. One hundred and twenty microliters of HBSS were added to 100 µL of the viral sucrose fraction, then two dilutions 10^−1^ and 10^−2^ were realized, and 1 mL was inoculated into 12.5 cm^2^ flasks with confluent monolayer cultures of both Vero and C6/36 cells and adsorbed for 2 h. They were maintained for 9–10 days and were examined daily for evidence of viral cytopathic effect (CPE). Cultures showing CPE were harvested for transmission electron microscopy.

## 3. Results

### 3.1. Mosquito Collection and Identification

A total of 4265 adult mosquitoes were collected during the study, including 657 from site 1, 496 from site 2, 293 from site 3, 1292 from site 4, 704 from site 5, and 823 from site 6 (Figure 1). Morphological identification indicated the presence of three different species: *Ae. albopictus* was the dominant species with 57% of the collected arthropods (2421/4265), followed by *Cx. pipiens* with 25% (1054/4265), and *Cs. longiareolata* with 18% (790/4265). The nature of the site influenced the composition of the collection. *Ae. albopictus* was the most abundant species collected in urban areas and, to a lesser extent, in peri-urban areas, whereas *Cx. pipiens* dominated in rural environments (Figure 1).

### 3.2. MALDI-TOF MS Analysis and Blind Tests

A total of 584 specimens were blindly submitted to MALDI-TOF MS to confirm the morphological identification. More than 92% (*n* = 542/584) mosquito legs MS spectra queried against the database conducted to relevant identification (LSVs > 1.8). Of these 542 specimens, 82.65% (*n* = 448/542) were identified with elevate log score values (LSVs) ranging between 2.001 to 2.593. MS spectra from solely 42 specimens did not succeed in reaching the LSV threshold value of 1.8 for confident identifications. This identification failing was mainly attributed to the lower quality of their MS spectra. The reduction in MS spectra intensity and diversity was attributed for the majority of them to the loss of several of their legs. These damaged specimens occurred during mosquito collection and transport.

**Figure 2:**
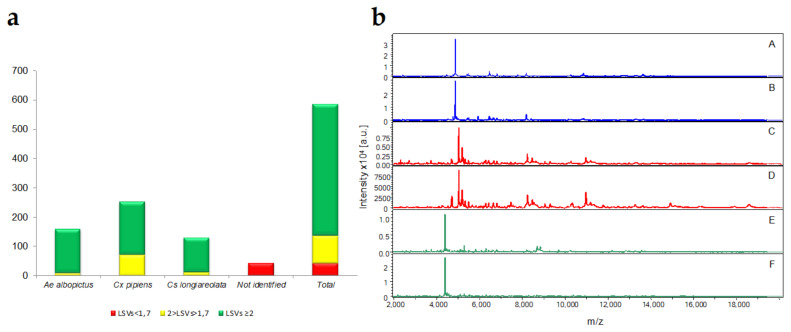
Mosquito species identification by MALDI-TOF MS-based on LSV ranges. (**a**) Results of MS spectra query for mosquito specimens tested blindly against the MS database were classified according to identification relevance based on LSVs. LSVs > 1.8, reliable identification (green); 1.8 > LSVs > 1.6, identification requiring manual validation (yellow); 1.6 > LSVs; corresponding to unreliable identification and considered as non-identified (red). (**b**) Representative MS spectra of three mosquito species were collected in this study. *Ae. albopictus* (A,B), *Cx. pipiens* (C,D), and *Cs. longiareolata* (E,F). Spectra analysis was performed using Flex analysis 3.3 software. a.u., arbitrary units; m/z, mass-to-charge ratio.

These 542 specimens were identified as *Ae. albopictus* (29.33%, *n* = 159/542), *Cx. pipiens* (46.67%, *n* = 253/542) and *Cs. longiareolata* (23.98%, *n* = 130/542) by MALDI -TOF MS (Figure 2a). The MS spectra comparison from different mosquito species revealed intra-species reproducibility and inter-species specificity using the Flex analysis software (Figure 2b).

### 3.3. TEM

Electron microscopy observations of the purified viral fractions showed the presence of viral particles with round structure in pools A (Supplementary File S3(i(a,b,c))) and B (Supplementary File S3 (ii(d,e))), with 200 nm and 80 nm diameter, respectively. No viral particles could be confidently identified in pool C.

### 3.4. Virome Composition Based on the Taxonomic Annotation of the Sequencing Reads

**Figure 3:**
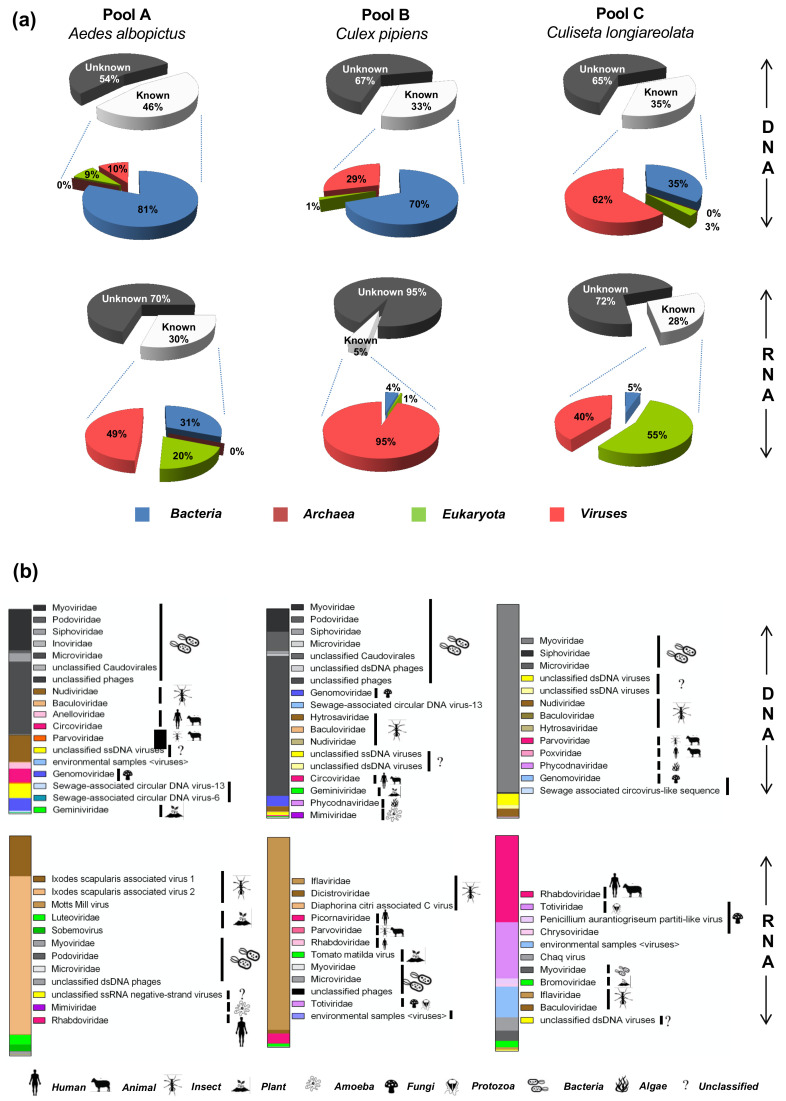
Taxonomic assignment of reads (**a**) Taxonomic annotation of the reads by MEGAN after Blast X search against the National Center for Biotechnology Information (NCBI) protein database using DIAMOND. (**b**) Relative abundance of viral families in mosquito metagenomes according to their target hosts.

RNA and DNA viromes of samples pool A, pool B, and pool C were sequenced using Illumina MiSeq technology. Sequencing statistics are presented in Table 2. Taxonomic assignment of reads identified 33–46% of DNA sequences and 5–30% of RNA sequences having similarities with known sequences in the database (Figure 3i). Sequences related to bacteria represented 81%, 70%, and 35% of the known sequences in the DNA viromes of pool A, pool B, and pool C, respectively (Figure 3a). Eukaryote-related sequences ranged from 1% to 9% for DNA viromes (Figure 3a) and 1% to 55% for RNA viromes. Very few reads were assigned to archaea in DNA and RNA viromes of pool A and DNA virome of pool C (Figure 3a). The reads assigned to viruses represented between 10% and 62% of the known reads for DNA viromes and from 40% to 95% of the known reads for RNA viromes (Figure 3a). Most viral reads of the DNA viromes were assigned to bacteriophages (60.76%, 89.59%, and 88.75% of total reads for pool A, pool B, and pool C, respectively) (Figure 3b). Insect-associated viral families (i.e., *Nudiviridae*, *Baculoviridae*, *Hytrosaviridae*, and *Parvoviridae*) were also identified in the DNA viromes of pools A, B, and C, at 14.07%, 2.81%, and 4.15%, respectively. The unclassified “*Ixodes scapularis* associated virus 2” and viruses within the *Iflaviridae* family represented the majority of viral reads of RNA viromes for pool A and pool B, at 71.78% and 91.74%, respectively. In contrast, in the RNA virome of pool C, we observed that 40.21% of total viral reads were assigned to the *Rhabdoviridae* family and 26.11% to the *Totiviridae* family, while insect viruses represented only 1.1%. Plant viruses (*Luteoviridae*, *Sobemoviridae*, and *Bromoviridae*) comprised 7.41%, 1.38%, and 3.00% of the total viral reads from pool A, pool B, and pool C RNA viromes, respectively, as well as 0.17% and 0.41% of total viral reads for pool A and pool B DNA viromes, respectively (Figure 3b). Several vertebrate and mammalian DNA viruses were also detected, such as *Anelloviridae*, in the pool A DNA virome, with an abundance of 2.97% of total viral reads, *Circoviridae* family was identified and represented 6.69% and 0.52% of total viral reads for pool A and pool B DNA viromes, respectively. The *Poxviridae* family was also detected in the pool C DNA virome with 0.05% of total reads. RNA viruses like the *Picornaviridae* family were detected in the pool B, RNA virome (Figure 3b). Reads assigned to giant viruses infecting algae and protozoan (*Phycodnaviridae* and *Mimiviridae*) represented 0.18% and 0.06% of DNA viromes of the pool B and C, respectively. Few DNA viruses were also identified in the RNA viromes (bacteriophages and amoeba-infecting giant viruses, representing 1.74%, 0.05%, and 4.78% of total viral reads for pool A, pool B, and pool C RNA viromes, respectively), possibly due to residual contamination of the RNA fraction by viral DNA (Figure 3b). Groups classified as environmental and unidentified viruses were also detected (Figure 3b).

### 3.5. Phylogenetic Analyses

#### 3.5.1. Viruses Associated with Animals

**Figure 4:**
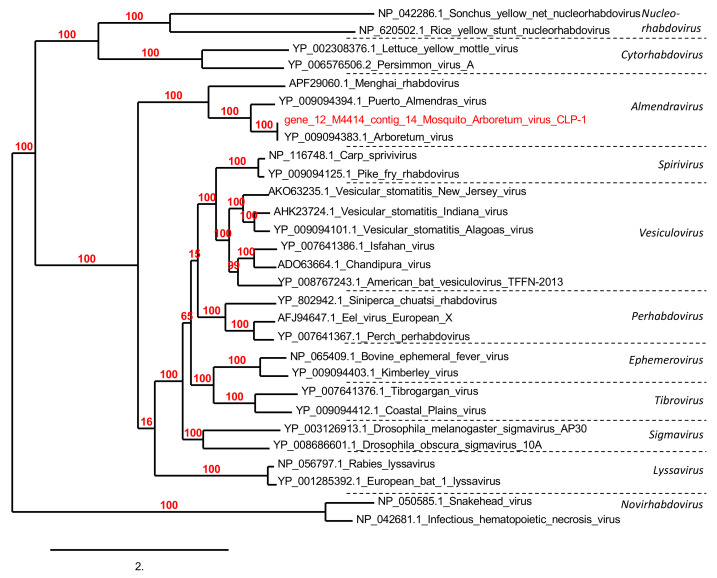
Phylogenetic analysis of the mosquito *Arboretum* virus CLP-1 compared to other *Rhabdoviridae* viruses.

Sequences (reads) assembled from the mosquito viromes had similarities to four families of animal viruses (Supplementary File S4). Among the animal viruses, *Rhabdoviridae* family was the most abundant, with a total of 54,197 assigned reads. More than 99% of these (54 117 reads) were identified in the *Cs. longiareolata* RNA virome. These reads assembled into six contigs ranging from 431 to 4126 bp in size and presenting a nucleotide identity of 33% to 96% with *Arboretum virus* within the proposed *Almendravirus* genus [43]; Gene prediction resulted in six ORFs ranging from 256 to 913 aa in size. Phylogenetic tree based on L protein using a sequence of 913 aa revealed that this virus is a *Rhabdovirus* close to *Arboretum virus* and *Almendras virus* isolated from *Psorophora albigenu* and *Ochlerotattus fulvus* mosquitoes in Peru [44] (Figure 4). This virus was named mosquito *Arboretum* virus CLP-1 (mAV-CLP-1). Analysis of the mAV-CLP-1 sequences indicated that it shared a similar genome organization with *Arboretum* virus and *Puerto Almendras* virus [44]. The partial genome features showed four of the five canonical *Rhabdovirus* structural protein genes (3′-N-P-M-G-L-5′) but lacked the M gene.

**Figure 5:**
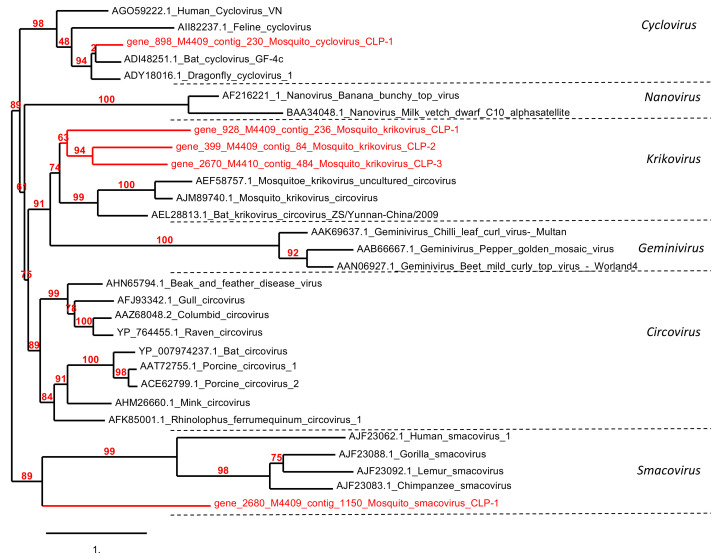
Phylogenetic analysis of mosquito *Cyclovirus* CLP-1, mosquito *Krikovirus* (CLP-1, 2, and 3), and mosquito *Smacovirus* CLP-1 compared to other *Circoviridae* viruses.

A total of 5179 reads assigned to *Circoviridae* family were also detected. Sequence assemblies allowed the reconstruction of 10 contigs ranging from 393 to 3 361 bp. Gene prediction identified 10 ORFs ranging from 60 aa to 342 aa in size. These ORFs have amino-acid identity mainly with *Bat circovirus* and *Bat cyclovirus*. Five annotated replication-associated proteins (Rep) were replaced in a phylogenetic tree of the *Circoviridae* family among the proposed *Cyclovirus* (*n* = 1), *Krikovirus* (*n* = 3), and *Smacovirus* (*n* = 1) genera. These were named mosquito *Cyclovirus* CLP-1, mosquito *Krikovirus* CLP (1, 2, and 3), and mosquito *Smacovirus* CLP-1. Except for mosquito *Krikovirus* CLP-3, which was found in the *Cx. pipiens* virome, all other identified viruses were detected from the *Ae. albopictus* virome (Figure 5).

A total of 2063 reads related to the *Anelloviridae* family were detected. These reads were assembled in three contigs of 563, 1759, and 2518 bp. These contigs presented a nucleotide identity of 32%, 31%, and 33% with the *Torque teno canis* virus, *Nayun tick torquevirus*, and *Torque teno canis virus*, respectively. They encode 3 ORFs sized of 138, 207 and 341 aa. One ORF presented amino-acid identity of 45% with *Torque tenocanis* virus and two with *Nayun tick torquevirus* with 37% and 31% identity, respectively.

Finally, within the DNA viromes obtained from *Cs. longiareolata* samples, 278 reads were assigned to the *Poxviridae* family. They were assembled in 25 contigs ranging from 313 to 2020 bp in size, from which 22 ORFs were predicted (48 aa to 421 aa). The ORFs matched with a wide range of poxviruses, belonging to both *Chordopoxvirinae* and *Entomopoxvirinae* sub-families. The *Chordopoxvirinae* sub-family was represented mostly by the *Avipoxvirus* genus, including the *Turkeypox* virus, *Fowlpox* virus, *Canarypox* virus, and *Pigeonpox* virus, with identities ranging from 42% to 75%. While the *Entomopoxvirinae* sub-family was mainly represented by the *Alpha entomopoxvirus* and *Beta entomopoxvirus,* including the three species *Mythimna separata entomopoxvirus*, *Anomalacuprea entomopoxvirus*, and *Melanoplussanguinipes entomopoxvirus*, with an amino-acid identity ranging from 27% to 69%.

#### 3.5.2. Protozoan and Fungus Viruses

**Figure 6:**
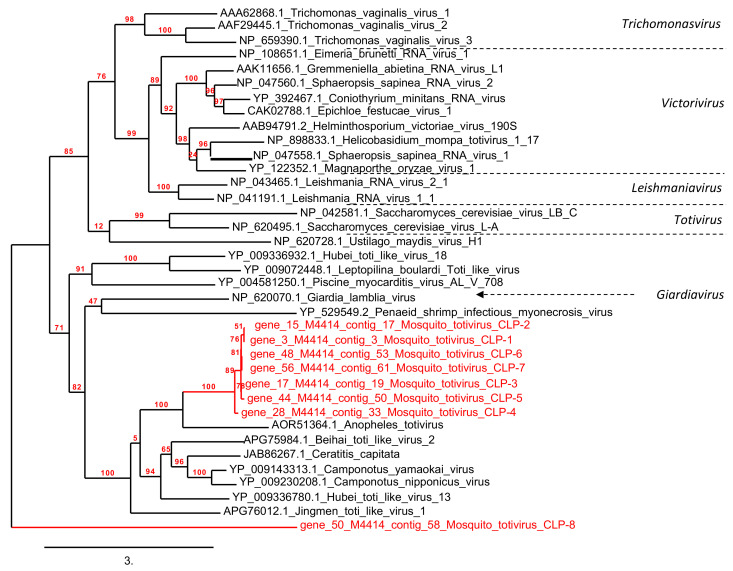
Phylogenetic analysis of mosquito *Totivirus* CLP-1 to CLP-8 compared to other *Totiviridae* viruses.

Sequences related to protozoan and fungus viral families were detected in the mosquito viromes, namely *Totiviridae* and *Genomoviridae* (Supplementary File S4). Viruses from the *Totiviridae* family represented a significant component within the RNA virome of the pool C *Cs. longiareolata* samples with a total of 35,144 reads. They were assembled in 20 contigs ranging from 482 to 1654 bp in size, from which 11 ORFs were predicted (240 aa to 410 aa). All ORFs matched with the viral RNA polymerase RdRp protein segment of *Anopheles totivirus*, with an amino-acid identity of 38% to 46%. Phylogenetic tree based on RdRp protein (>300 aa) revealed that the majority were grouped with an uncharacterized clade close to an *Anopheles totivirus* isolated from *Anopheles* mosquitoes in Western Africa [45] (Figure 6).

The DNA viromes of *Ae. albopictus, Cx. pipiens*, and *Cs. longiareolata* contained a total of 9612 reads related to the *Genomoviridae* family; they were assembled in five contigs ranging from 388 to 2335 bp. These contigs presented a nucleotide identity of 49% to 69% with Pacific flying fox feces associated *gemycircularvirus*-8, Sewage-associated *gemycircularvirus* 4 and 5, and Human plasma-associated *gemycircularvirus*. They encoded 11 ORFs sized from 88 to 258 aa witch presented amino-acid identity ranging from 39% to 70% with: Pacific flying fox feces associated *gemycircularvirus*-8, *Poecile atricapillus* GI tract-associated *gemycircularvirus*, Odonata associated *gemycircularvirus*-1 and 2, Pacific flying fox feces associated *gemycircularvirus*-10, Sewage-associated *gemycircularvirus* 5, and Human plasma-associated *gemycircularvirus* and Feces associated *gemycircularvirus* 17 and 22.

#### 3.5.3. Arthropod Viruses

Sequences (reads) assembled from the mosquito viromes had similarities to five families of arthropod viruses, namely *Iflaviridae*, *Parvoviridae*, *Baculoviridae, Nudiviridae* and *Hytrosaviridae*, and the unclassified *Ixodes scapularis* associated virus 2 (Supplementary File S4).

**Figure 7:**
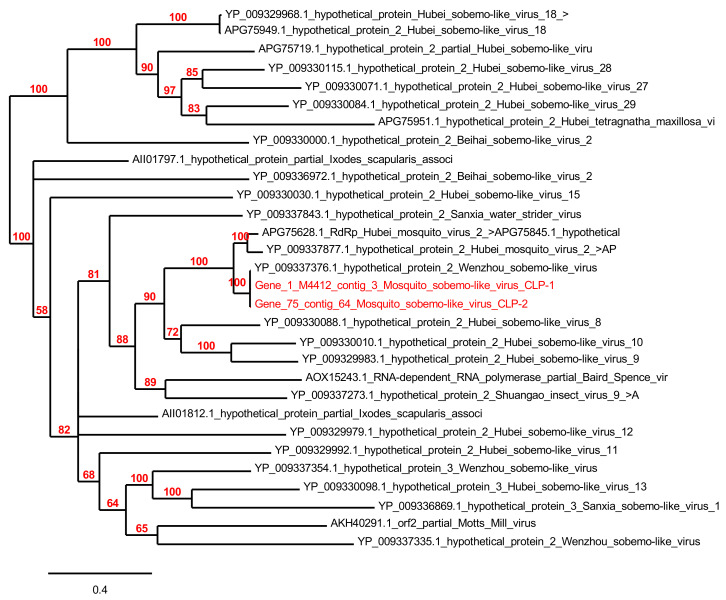
Phylogenetic analysis of mosquito *Somebo*-like virus CLP-1 and 2 compared to other Sobemo viruses.

Sequences matching with “*Ixodes scapularis* associated virus 2” were particularly abundant within the RNA virome of pool A (*Ae. albopictus* samples), with a total of 95,536 reads. They were assembled in three contigs ranging from 679 to 2529 bp in size, from which three ORFs were predicted (104 aa to 266 aa). Two of the ORFs matched with the hypothetical protein 2 of Wenzhou sobemo-like virus 4, with 100% identity, and one matched with the hypothetical protein 1 of Wenzhou sobemo-like virus 4, with 95% identity. Phylogenetic tree based on the hypothetical protein revealed that our viruses named mosquito *sobemo*-like virus CLP-1 and CLP-2 were close to other *sobemo*-like viruses (unclassified RNA viruses) isolated from mosquitoes in China [25] (Figure 7).

A total of 71,471 reads from the *Cx*. *pipiens* and *Cs*. *longiareolata* pools assigned to the *Iflaviridae* family were detected in the RNA viromes. Sequence assemblies allowed the reconstruction of five contigs ranging from 481 to 9767 bp, matching with a nucleotide identity of 38% to 61% to the Hubei picorna-like virus 35 and 41, and the *Nasonia vitripennis* virus. Gene prediction identified six ORFs of 101 to 3126 aa in size that matched mainly with hypothetical protein from the Hubei picorna-like virus 35 with 31% to 61% identity.

A total of 938 reads were assigned to *Parvoviridae* within the DNA virome of the pools A and C (*Ae. albopictus* and *Cs. longiareolata* samples). They were assembled in 17 contigs ranging from 305 to 3827 bp in size, from which 19 ORFs were predicted (97 aa to 618 aa). The majority of the ORFs had similarities to insect densoviruses with an identity of 26% to 100% (Supplementary File S4), including *Acheta domestica* mini ambidensovirus, *Blattella germanica* densovirus-like virus, *Pseudoplusia includens* densovirus, *Diaphorina citri* densovirus, *Mythimna loreyi* densovirus, *Diatraea saccharalis* densovirus and *Junonia coenia* densovirus, as well as the bird-like *Parus major* densovirus.

A total of 2320 reads related to *Baculoviridae* family were detected in the DNA viromes of the three mosquito species. These sequences were assembled into four contigs sized 903 to 10 488 bp leading to the prediction of seven ORFs ranging from 97 to 417 aa. The ORFs shared a range of 26%–53% of identity with many baculoviruses belonging to different genera: *Autographa californica* nucleopolyhedrovirus, *Pseudaletia unipuncta* granulovirus, *Mamestra configurata* nucleopolyhedrovirus B, *Leucania separata* nucleopolyhedrovirus, as well as an Ascovirus *Heliothis virescens ascovirus 3f.*

A total of 9169 reads were assigned to *Nudiviridae* family within the DNA virome of pools A and B (*Ae. albopictus* and *Cx. pipiens* samples). They were assembled in 40 contigs ranging from 357 to 9000 bp in size, from which 47 ORFs were predicted (55 aa to 544 aa). The ORFs matched with different species with a range of 31% to 69% identity (Supplementary File S4).

Finally, 2997 reads were assigned to *Hytrosaviridae* family within the viromes of the *Cx. pipiens* and *Cs. longiareolata* samples. They were assembled in 38 contigs ranging from 305 to 7355 bp in size, from which 15 ORFs were predicted (83 aa to 581 aa). The ORFs matched with *Musca domestica* salivary gland hypertrophy virus and *Glossina pallidipes* salivary gland hypertrophy virus.

#### 3.5.4. Plant Viruses

Sequences with similarities to plant virus families were notably identified in the mosquito viromes (Figure 3 and Supplementary File S4). Namely, *Luteoviridae*, Sobemovirus, *Bromoviridae*, *Tomato matilda* virus and *Geminiviridae.* However, the majority of these groups were represented just by a few reads detected in the RNA viromes such as *Luteoviridae*, Sobemovirus, and *Tomato matilda* virus. A total of 4037 reads were assigned to *Bromoviridae* family within the RNA virome of the *Cs. longiareolata* sample. They were assembled in one contig of 1481 bp leading to the prediction of one ORF sized 441 aa. This ORF matched with 86% identity with Blackberry chlorotic ringspot virus.

For the *Geminiviridae* family, two ORFs, coding for 185 and 186 aa putative proteins, were predicted. Both sequences matched with putative replication initiation protein (*Lytechinus variegatus* variable sea urchin associated circular virus) with 36% and 37% identity.

#### 3.5.5. Amoeba and Algae Viruses (Giant Viruses)

Interestingly, sequences related to the *Mimiviridae* family were detected in the DNA virome of *Ae. albopictus.* A contig of 665 bp matched with *Moumouvirus monve* with 38% nucleotide identity. ORF prediction led to a sequence of 221 aa with 38% similarity with an integrase zinc-binding and helicase domain-containing protein of *Moumouvirus monve*.

### 3.6. Virus Isolation on Vero and C6/36 Cells

After inoculation of the sucrose fraction (dilution 1/10) containing purified viral particles from the *Ae. albopictus* pool into flask cultures of Vero cells, a cytopathic effect (CPE) was observed after 9–10 days. The infected Vero cells were dead and formed holes in the cellular mat (Supplementary File S5). However, no virus particles could be detected with TEM. No CPE was observed in Vero cells infected with dilution 1/100. Isolation on C6/36 did not succeed; no viral form was observed.

## 4. Discussion

In this study, we performed viral metagenomics on pooled *Ae. albopictus, Cx. pipiens* and *Cs. longiareolata* collected from the French Riviera region. Since the introduction of the invasive Asian mosquito *Ae. albopictus* in 2004 on this area [8,9], several imported and autochthonous cases of Dengue, Chikungunya, and Zika viruses were identified [11] in different districts: two cases of autochthonous Dengue fever and Chikungunya fever were reported in Nice and Fréjus respectively, in September 2010 [46,47]. In 2014, an outbreak of 12 autochthonous Chikungunya cases due to a strain imported with a traveler returning from Cameroon was detected in a district of Montpellier [48]. Cases are still being identified now. In the present study, MALDI-TOF MS technology was used for the identification of the adult mosquito population. This proteomic tool has emerged in the field of medical entomology, including the identification of mosquitoes [29,33] collected in the field as well as other arthropods families [49]. Using this strategy, rapid identification of specimens collected during the week was performed, allowing us to save time and to avoid cumulative manipulation of the specimens, which had to be moved rapidly at −80 °C for the virome exploration and isolation assays.

The monitoring of mosquito populations during these five months showed variations in density and species from one site to another. High prevalence of *Ae. albopictus* species was observed especially close to schools and individual houses situated in the center of the village. This could be explained by the fact that the area is endemic to the tiger mosquito, which has invaded it since 2011 and by the anthropophilic character of *Ae. albopictus*. Indeed, *Ae. albopictus* originated in Asian forests and commonly found in rural and peri-urban settings is currently occurring in highly dense urban areas and rapidly extended from one city to another [50].

*Cx. pipiens* is the second most abundant species collected. *Cx. pipiens* were found in all prospected sites, but higher abundance was found in traps close to ranch housing horses. *Cx. pipiens* is a species with two forms with an intermediate (hybrid) form feeding on both mammals (humans and equids) and birds making it a bridge for zoonotic agent transmission. Finally, *Cs. Longiareolata,* a bird-seeking mosquito was found abundant in the most rural area. Both *Ae. albopictus* and *Cx. pipiens* mosquitoes are incriminated as competent vectors of several human arboviruses.

In this study, we reported an extensive description of the DNA and RNA viral communities of three mosquito species collected in Cuges-les-Pins. Analysis of the taxonomic assignment of reads revealed a high proportion of sequences related to viruses which can reflect the database increment with RNA and DNA viral sequence these last years. Although the three mosquito species were collected at the same location sites during the same week using the same traps, the six RNA and DNA metagenomes have highly divergent profiles in terms of relative abundance and composition of viral communities [24,34,51].

The DNA viromes of *Ae. albopictus*, *Cx. pipiens*, and *Cs. longiareolata* were largely dominated by sequences related to bacteriophages from the *Myo viridae*, *Podo viridae*, *Sipho viridae*, and *Microviridae* families and unclassified phages. These sequences may derive from phages infecting the bacterial microflora of the mosquitoes or that of the plant/animal hosts on which they fed upon. In that sense, sequences related to mammal viruses belonging to the *Rhabdoviridae*, *Circoviridae*, *Anelloviridae*, and *Poxviridae* families were also detected in the DNA and RNA viromes and may reflect the blood meal of the mosquitoes. These families were already identified in mosquito viromes [19].

The *Rhabdoviridae*-related sequences consisted mainly of the RNA virome of *Cs. longiareolata*. *Rhabdoviridae* is a family of negative-sense RNA viruses, which includes 19 genera according to the International Committee on Taxonomy of Viruses (ICTV). Among them, the rabies virus, which causes fatal disease in humans and continues to kill an estimated 40,000–70,000 people yearly [52]. Rabies virus has a tropism for mammals, but other rhabdoviruses can infect fish, insects, and plants [51]. Indeed, novel rhabdoviruses continue to be detected, especially in field-collected mosquitoes [53,54,55]. The mosquito *Arboretum virus* CPL-1 strain described here is closely related to *Rhabdoviridae* viruses isolated from different mosquito species in the Americas [43,44]. These results suggest that these viruses are probably insect-specific and present a wide geographic distribution. *Circoviridae* is a single-stranded circular DNA (ssDNA) genomes viral family composed of two genera *Circovirus* and *Cyclovirus*. *Porcine circovirus* 1 and 2 belonging to the *Circovirus* genus have been extensively documented to replicate in a mammal, cause a variety of clinical symptoms with significant economic impact [56]. In this study, novel *Circoviridae* were identified belonging to the proposed *Cyclovirus*, *Krikovirus* and *Smacovirus* genera. So far, the *Krikovirus* genus was only composed of viruses identified in mosquitoes and bat feces [57]. Based on phylogenetic analyses, the mosquito *Krikoviruses* identified in the *Ae. albopictus* and *Cx. pipiens* mosquito pools of this study form a distinct clade in the proposed *Krikovirus* genus.

*Anelloviridae* is a family of negative sense ssDNA viruses. In the present study, the sequences had low similarities with *Torque tenocanis virus* (genus *Thetatorquevirus*) isolated from the sera of domestic pigs and dogs [58] and *Nayun tick torquevirus* isolated from *Rhipicephalus* ticks in China [23], suggesting that collected mosquitoes may feed on dogs and Anelloviruses are widely spread. Anelloviruses have already been detected in field-collected mosquitoes [51,59].

*Poxviridae* is a family of large dsDNA viruses divided into two sub-families *Chordopoxvirinae* and *Entomopoxvirinae* composed of 11 and 3 genera, respectively. It is a group that can infect a large number of animals causing virulent pathologies like variola in humans [60]. A few years ago, a recombinant canarypox virus was used as a vaccine and protected cats, dogs, and horses against West Nile Virus, which is transmitted by mosquitoes [61,62,63,64]. A poxvirus causing lumpy skin disease was proved to be mechanically transmissible by *Ae*. *aegypti* from infected to susceptible cattle [65]. More recently, in southern England, the myxoma virus was detected in the *Anopheles maculipennis* complex [66]. Likewise, Yoka poxvirus was isolated almost four decades ago from a mosquito pool in the Central African Republic [67]. These studies suggest that mosquitoes might transmit poxviruses, although carriage does not mean vectorial competence. Recently, a case of a previously unknown poxvirus rash illness in a renal transplant patient was reported. The identified virus was closely related to the Yoka virus [68].

*Totiviridae* was the second most abundant family composing the RNA virome of *Cs. longiareolata*. To date, according to ICTV, the *Totiviridae* family comprises five genera formally recognized: *Giardiavirus*, *Leishmaniavirus*, *Totivirus*, *Trichomonasvirus*, and *Victorivirus*. These genera include viruses infecting fungi and protozoa. Here, the sequences clustered with *Anopheles totivirus*, a virus recently isolated in *Anopheles* mosquitoes in Africa [45]. In recent years, several new *Totiviruses* have been identified in different mosquito species [69,70,71].

*Genomoviridae* is a new family of ssDNA viruses; it contains one genus *Gemycircularvirus* with one recognized virus species, *Sclerotinia gemycircularvirus* 1, which was isolated from *Sclerotinia sclerotiorum* a plant-pathogenic fungus [72]. Sequences related to the *Genomoviridae* family were detected in four mosquito viromes collected in China [51]. Although we cannot rule out the possibility that these viruses infect commensal fungi or protists constituting the microbiome of mosquitoes as previously underlined [45], the fact that these viruses are exclusively associated with arthropods, including mosquitoes, that are circulating in very different biotopes worldwide [53,73] strongly suggests that these represent novel Insect-specific viruses (ISVs). ISVs are viruses that naturally infect mosquitoes and replicate in mosquito cells in vitro but do not appear to replicate in vertebrate cells or infect humans or other vertebrates [74]. The RNA viromes of the three mosquito species were largely different in terms of viral composition. Indeed, the virome of *Ae. albopictus* was dominated by sequences matching almost a unique virus identified as *Ixodes scapularis* virus 2 after annotation of the reads with DIAMOND/MEGAN. However, further blast P analysis of the ORFs against the whole NCBI revealed a similarity of 95%–100% with the recently described Wenzhou sobemo-like virus 4 [25]. This virus was identified by transcriptomics from a pool of mosquitoes that included five *Ae. albopictus* individuals collected in China [25]. The very high percentage of sequence identity between the two viral sequences detected from mosquitoes as far as China and France, suggests a broad distribution of this virus in mosquitoes that is not influenced by geographical location. The RNA virome of *Cx. pipiens* was also largely dominated by sequences assigned to the unique *Iflaviridae* family. Blast P analysis of the ORFs against the NCBI database showed a best-blast hit with Hubei picorna-like virus 35, a virus identified in an odonata mix from the Hubei region in China [25], but the low percentage of identity (38% to 60%) suggest that these sequences detected in *Cx. pipiens* represent novel picorna-like virus. *Parvoviridae* is an ssDNA viruses family composed of two sub-families *Parvovirinae* and *Densovirinae*, regrouping eight and six genera, respectively, according to the ICTV. Parvoviruses were already detected in wild-caught mosquitoes worldwide [19,50,58]. In this study, many ORFs showed low amino-acid identities to known insect densoviruses suggesting the presence of many novel insect viruses in *Ae. albopictus* and *Cs. longiareolata* mosquito species. However, the presence of *Parus major* densovirus in mosquitoes probably attributed to the blood-sucking of the mosquitoes from viremic birds.

*Baculoviridae* is a family of dsDNA viruses including four genera according to ICTV *Alphabaculovirus*, *Betabaculovirus*, *Deltabaculovirus*, and *Gammabaculovirus*. Members of this family infect larvae of the insect orders Lepidoptera, Hymenoptera, and Diptera [75] exclusively. *Baculoviridae* related sequences were already detected in *Culex quinquefasciatus* and *Culex tritaeniorhynchus* collected from Kenya and China [76]. *Hytrosaviridae* is a new family of insect dsDNA viruses [77], grouping two genera *Glossinavirus* and *Muscavirus* [78]. Actually, two species are recognized *Glossina pallidipes* salivary gland hypertrophy virus (SGHV) and *Musca domestica* SGHV. To our knowledge, it is the first time that *Hytrosaviridae*-related sequences were detected in mosquito viromes. Interestingly, *Mimiviridae* related sequences were detected in this study. The sequences had low similarities (38%) with *Moumouvirus Monve. Moumouvirus* represents the sublineage B of the *Mimiviridae* family [79]. As giant viruses infect amoebae and algae, the virus might come from mosquito larval habitat.

Isolation is mandatory to take a step further in the characterization of these newly described viruses. Indeed, additional in vivo and in vitro infection experiments would help to fully understand their biology. In this study, isolation of viral particles on C6/36 and Vero cells was unsuccessful, although a CPE was observed in the last one. Such attempts should be conducted again on these and other relevant cell lines to demonstrate the ability of these viruses to replicate in mammalian cells/mosquito cell lines or both, as the first step to decipher the role of these viruses *in natura.*

## 5. Conclusions

This study contributes to the knowledge of the repertoire of viruses associated with mosquitoes collected in France and highlights the potential pool of unknown viruses yet to be characterized, which could either represent emerging human pathogens. Using viral metagenomics, we detected one novel rhabdovirus, five novel circoviruses, eight novel totiviruses, and two sobemo-like viruses. Further in-depth studies are essential to characterize these newly described mosquito viruses and to distinguish mosquito-specific viruses from those that may present a serious risk of human infection. It would also be very interesting to carry out seasonal monitoring to determine if viral population dynamics of each mosquito species are modified according to the season and consequently by the influence of the environmental factors.

## Figures and Tables

**Table 1:** Pools for virome preparation.

	Pool A *Aedes albopictus*	Pool B *Culex pipiens*	Pool C *Culiseta longiareolata*
Site 1	20	8	20
Site 2	20	18	20
Site 3	20	18	16
Site 4	20	31	24
Site 5	8	11	12
Site 6	12	14	8
Total	100	100	100

**Table 2:** Virome dataset statistics.

	Pool A *Ae. albopictus*		Pool B *Cx. pipiens*		Pool C *Cs. longiareolata*	
	DNA	RNA	DNA	RNA	DNA	RNA
Raw reads (R1)	2,120,986	1,453,981	1,547,071	1,806,087	2,581,494	1,405,593
Trimmed reads (R1)	2,066,242	1,410,512	1,493,456	1,752,390	2,490,631	1,316,476
Assigned reads (R1)	972,296	430,138	503,057	87,106	901,150	391,087
Contigs	13,261	3789	4873	342	9440	1501
Average contig length (bp)	794	530	1083	570	768	669
ORFs	15,141	3674	9071	407	9202	1653

## Data Availability

The data presented in this study are openly available in the Sequence Read Archive (SRA) of the NCBI under the BioProject with accession code PRJNA723282, ID number 723282 at (https://www.ncbi.nlm.nih.gov/Traces/study/?acc=PRJNA723282).

## Supplementary Materials

The following are available online at https://www.mdpi.com/article/10.3390/v13050768/s1. Supplementary File S1. Schematic of the workflow for mosquito handling: from collection to bioinformatic analysis of metagenomes. Supplementary File S2. Details on the composition of pools for virome preparation. Supplementary File S3. Example of virus-like particles observed in the purified viral fraction by transmission electron microscopy after negative staining. (i(a,b,c)) pool A, (ii(d,e)) pool B. Supplementary File S4. Classification of animal, protozoan, insect, plant, and giant virus sequences present in the DNA and RNA mosquito viromes. Supplementary File S5. Cytopathology on Vero cells of the viruses of the pool A. (A, B) Control (non-infected) Vero cells. (C, D) CPE seen in Vero cells infected with dilution 1/10, 9 (C), and 10 (D) days after sucrose viral fraction infection; cells are dead and forming holes in the cellular mat (arrows). (E, F) Vero cells infected with dilution 1/100, no CPE was observed.
