# Supplementary material for: Virome Diversity among Mosquito Populations in a Sub-Urban Region of Marseille, France
Source: Viruses. 2021 Apr 27;13(5):768. doi: 10.3390/v13050768 (PMC8145591; doi:10.3390/v13050768)
Supplement: Supplementary file 1 [file viruses-13-00768-s001.zip › Supplementary_File_S1.pptx]

## Slide 1
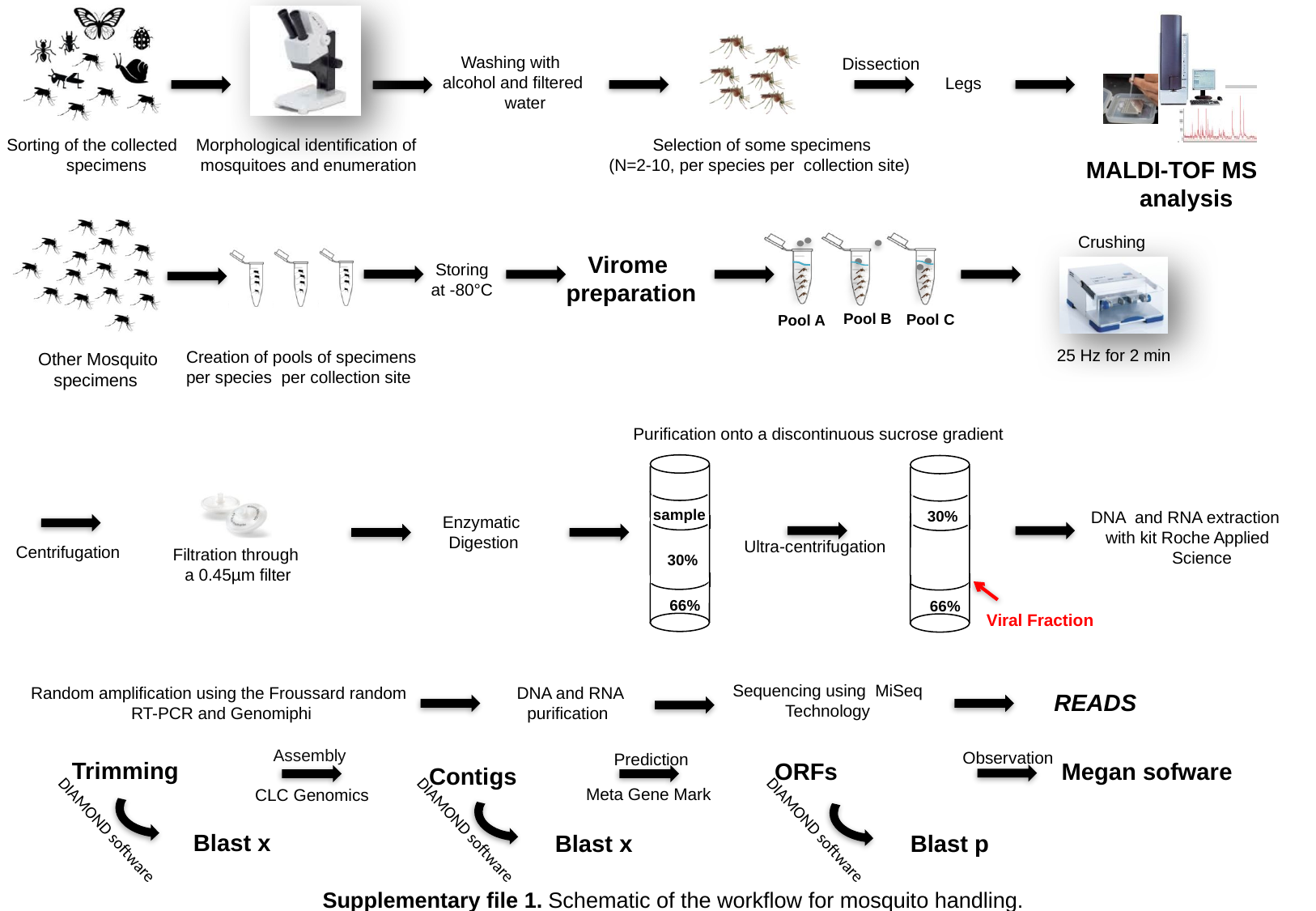

Washing with
 alcohol and filtered water
Dissection
Legs
Sorting of the collected specimens
Morphological identification of
 mosquitoes and enumeration
Selection of some specimens
(N=2-10, per species per collection site)
MALDI-TOF MS analysis
Crushing
Pool B
Pool C
Pool A
Virome
preparation
Storing
at -80°C
25 Hz for 2 min
Creation of pools of specimens
per species per collection site
Other Mosquito
specimens
Purification onto a discontinuous sucrose gradient
sample
30%
66%
30%
66%
Viral Fraction
DNA and RNA extraction
 with kit Roche Applied Science
Enzymatic
Digestion
Ultra-centrifugation
Centrifugation
Filtration through
a 0.45µm filter
Sequencing using MiSeq
Technology
 Random amplification using the Froussard random RT-PCR and Genomiphi
 DNA and RNA
purification
READS
Assembly
Observation
Prediction
Trimming
ORFs
Megan sofware
 Contigs
Meta Gene Mark
CLC Genomics
DIAMOND software
DIAMOND software
DIAMOND software
 Blast x
 Blast x
 Blast p
Supplementary file 1. Schematic of the workflow for mosquito handling.
