# Supplementary material for: Virome Diversity among Mosquito Populations in a Sub-Urban Region of Marseille, France
Source: Viruses. 2021 Apr 27;13(5):768. doi: 10.3390/v13050768 (PMC8145591; doi:10.3390/v13050768)
Supplement: Supplementary file 1 [file viruses-13-00768-s001.zip › Supplementary_File_S2.pdf]

Supplementary file 2. Details on the composition of pools for virome preparation.

	Pool A ***Aedes albopictus***		
	**Collection week**	***Specimens Number***	**Date of collection**
**Site 1**	23	1	06/06/2016
	24	1	06/13/2016
	25	1	06/20/2016
	26	1	06/28/2016
	29	1	07/18/2016
	30	1	07/25/2016
	31	1	08/01/2016
	32	1	08/08/2016
	33	1	08/15/2016
	34	2	08/22/2016
	35	2	08/28/2016
	36	1	09/05/2016
	37	1	09/12/2016
	38	1	09/19/2016
	39	1	09/26/2016
	40	1	10/03/2016
	41	1	10/17/2016
	42	1	11/02/2016
**Total**		20	
**Site 2**	23	1	06/06/2016
	24	1	06/13/2016
	26	1	06/28/2016
	27	1	07/04/2016
	29	1	07/18/2016

	30	1	07/25/2016
	31	2	08/01/2016
	32	2	08/08/2016
	33	2	08/15/2016
	34	1	08/22/2016
	35	1	08/28/2016
	36	2	09/05/2016
	37	1	09/12/2016
	38	1	09/19/2016
	40	1	10/03/2016
	41	1	10/17/2016
**Total**		20	
	24	1	06/13/2016
	25	1	06/20/2016
	26	1	06/28/2016
	27	1	07/04/2016
	30	1	07/25/2016
	31	1	08/01/2016
	32	2	08/08/2016
**Site 3**	33	2	08/15/2016
	34	1	08/22/2016
	35	2	08/28/2016
	36	1	09/05/2016
	37	1	09/12/2016
	38	1	09/19/2016
	39	1	09/26/2016
	40	1	10/02/2016

	41	1	10/17/2016
	43	1	11/02/2016
**Total**		20	
	24	1	06/13/2016
	26	1	06/28/2016
	27	1	07/04/2016
	28	1	07/11/2016
	29	1	07/18/2016
	31	1	08/01/2016
	33	2	08/15/2016
**Site 4**	34	1	08/22/2016
	35	3	08/28/2016
	36	2	09/05/2016
	37	1	09/12/2016
	38	1	09/19/2016
	39	1	09/26/2016
	40	2	10/02/2016
	41	1	10/17/2016
**Total**		20	
	26	1	06/28/2016
	28	1	07/11/2016
	30	1	07/25/2016
	32	1	08/08/2016
	34	1	08/22/2016
	38	1	09/19/2016
	40	1	10/03/2016
	42	1	10/17/2016

**Total**		8	
**Site 6**	29	1	07/18/2016
	31	1	08/01/2016
	33	1	08/15/2016
	35	2	08/29/2016
	36	2	09/05/2016
	37	2	09/12/2016
	39	1	09/26/2016
	41	1	10/10/2016
	43	1	11/02/2016
**Total**		12	
Pool B ***Cx. pipiens***			
	**Collection week**	***Specimens Number***	**Date of collection**
**Site 1**	28	1	07/11/2016
	29	1	07/18/2016
	30	1	07/25/2016
	31	1	08/01/2016
	32	2	08/08/2016
	33	1	08/15/2016
	35	1	08/28/2016
**Total**		8	
**Site 2**	23	2	06/06/2016
	24	1	06/13/2016
	26	2	06/28/2016
	27	3	07/04/2016
	28	2	07/11/2016
	30	1	07/25/2016

	33	2	08/15/2016
	35	2	08/28/2016
	38	1	09/19/2016
	39	1	09/26/2016
	41	1	10/17/2016
**Total**		18	
**Site 3**	23	2	06/06/2016
	24	2	06/13/2016
	25	2	06/20/2016
	27	1	07/04/2016
	32	1	08/08/2016
	33	3	08/15/2016
	34	2	08/22/2016
	35	4	08/28/2016
	40	1	10/02/2016
**Total**		18	
**Site 4**	23	1	06/06/2016
	24	2	06/13/2016
	25	2	06/20/2016
	26	2	06/28/2016
	27	1	07/04/2016
	28	3	07/11/2016
	29	1	07/18/2016
	30	2	07/25/2016
	31	2	08/01/2016
	32	2	08/08/2016
	33	3	08/15/2016

	35	3	08/28/2016
	37	1	09/12/2016
	38	1	09/19/2016
	39	3	09/26/2016
	40	2	10/02/2016
**Total**		31	
	26	2	06/28/2016
	27	1	07/04/2016
	28	2	07/11/2016
	29	1	07/18/2016
**Site 5**	30	1	07/25/2016
	32	1	08/08/2016
	33	1	08/15/2016
	40	1	10/03/2016
	43	1	11/02/2016
**Total**		11	
	26	1	06/28/2016
	27	2	07/04/2016
	28	1	07/11/2016
	29	1	07/18/2016
	30	1	07/25/2016
**Site 6**	31	1	08/01/2016
	32	1	08/08/2016
	33	1	08/15/2016
	34	1	08/22/2016
	35	1	08/29/2016
	37	1	09/12/2016

	38	1	09/19/2016
	40	1	10/03/2016
**Total**		14	

Pool C ***Cs. longiareolata***			
	**Collection week**	***Specimens Number***	**Date of collection**
**Site 1**	23	2	06/06/2016
	24	3	06/13/2016
	25	2	06/20/2016
	28	1	07/11/2016
	29	1	07/18/2016
	31	2	08/01/2016
	34	1	08/22/2016
	35	2	08/28/2016
	36	2	09/05/2016
	37	1	09/12/2016
	40	2	10/03/2016
	41	1	10/17/2016
**Total**		20	
**Site 2**	26	2	06/28/2016
	27	2	07/04/2016
	28	1	07/11/2016
	29	2	07/18/2016
	30	2	07/25/2016
	31	2	08/01/2016
	32	1	08/08/2016
	33	2	08/15/2016
	34	2	08/22/2016

	35	1	08/28/2016
	36	1	09/05/2016
	38	1	09/19/2016
	40	1	10/03/2016
**Total**		20	
	24	2	06/13/2016
	25	3	06/20/2016
	26	2	06/28/2016
	29	1	07/18/2016
	31	1	08/01/2016
**Site 3**	34	1	08/22/2016
	36	1	09/05/2016
	38	1	09/19/2016
	40	1	10/02/2016
	41	2	10/17/2016
	43	1	11/02/2016
**Total**		16	
	24	1	06/13/2016
	25	2	06/20/2016
	26	3	06/28/2016
	27	3	07/04/2016
	28	2	07/11/2016
**Site 4**	29	2	07/18/2016
	30	3	07/27/2016
	33	2	08/15/2016
	37	1	09/12/2016
	38	2	09/19/2016

	39	1	09/26/2016
	40	1	10/02/2016
	43	1	10/17/2016
**Total**		24	
	26	2	06/28/2016
	27	1	07/04/2016
	28	1	07/11/2016
	30	1	07/25/2016
**Site 5**	31	1	08/01/2016
	38	2	09/19/2016
	40	1	10/03/2016
	41	2	10/10/2016
	42	1	10/17/2016
**Total**		12	
	26	1	06/28/2016
	27	3	07/04/2016
**Site 6**	28	1	07/11/2016
	30	1	07/25/2016
	32	1	08/08/2016
	41	1	10/10/2016
**Total**		8	
