# Supplementary material for: Virome Diversity among Mosquito Populations in a Sub-Urban Region of Marseille, France
Source: Viruses. 2021 Apr 27;13(5):768. doi: 10.3390/v13050768 (PMC8145591; doi:10.3390/v13050768)
Supplement: Supplementary file 1 [file viruses-13-00768-s001.zip › Supplementary_File_S5.pptx]

## Slide 1
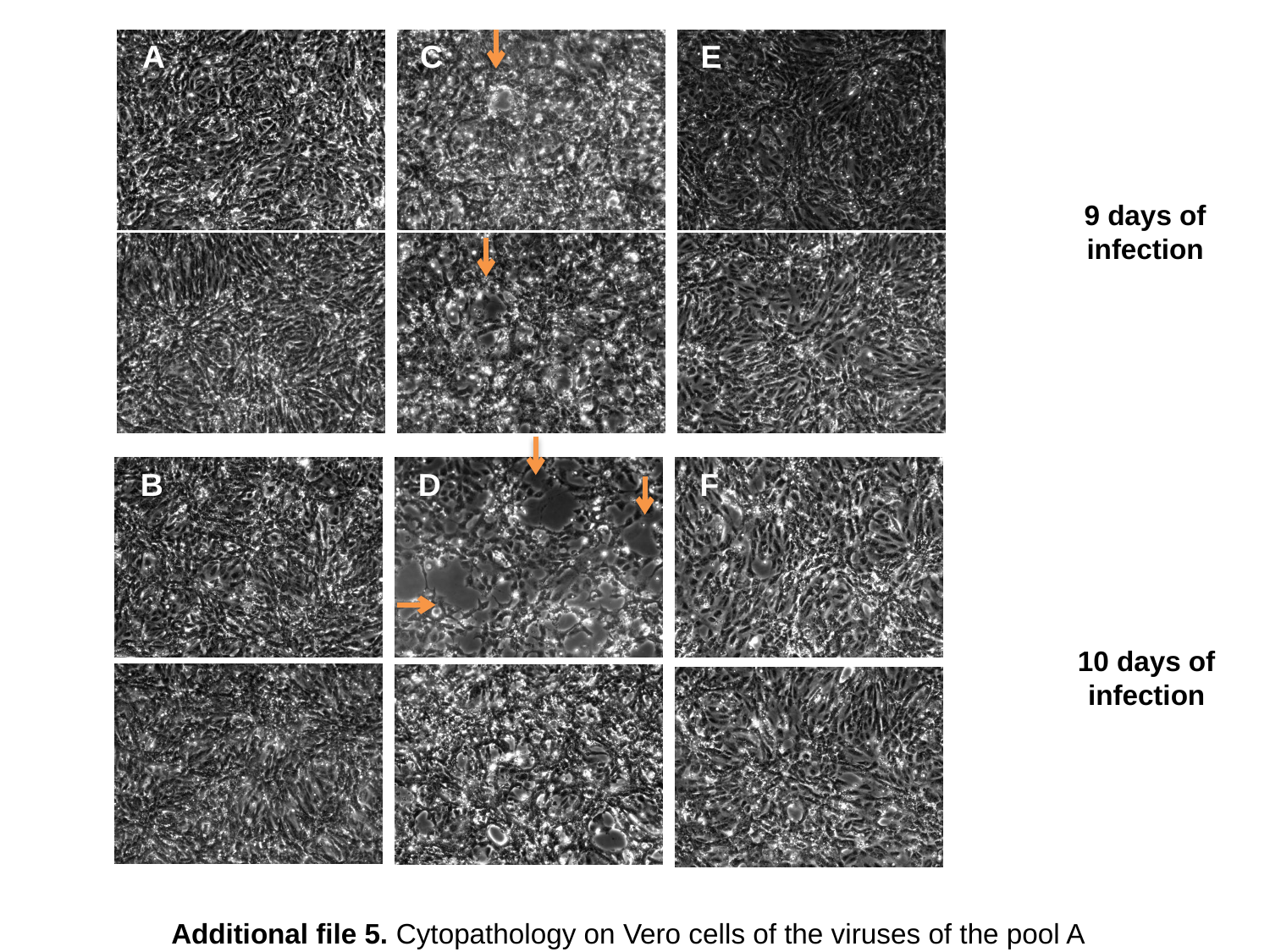

A
C
E
9 days of infection
P0J9
B
D
F
10 days of infection
Additional file 5. Cytopathology on Vero cells of the viruses of the pool A
