# Supplementary figures and images for: Virome Diversity among Mosquito Populations in a Sub-Urban Region of Marseille, France
Source: Viruses. 2021 Apr 27;13(5):768. doi: 10.3390/v13050768 (PMC8145591; doi:10.3390/v13050768)

## Slide 1
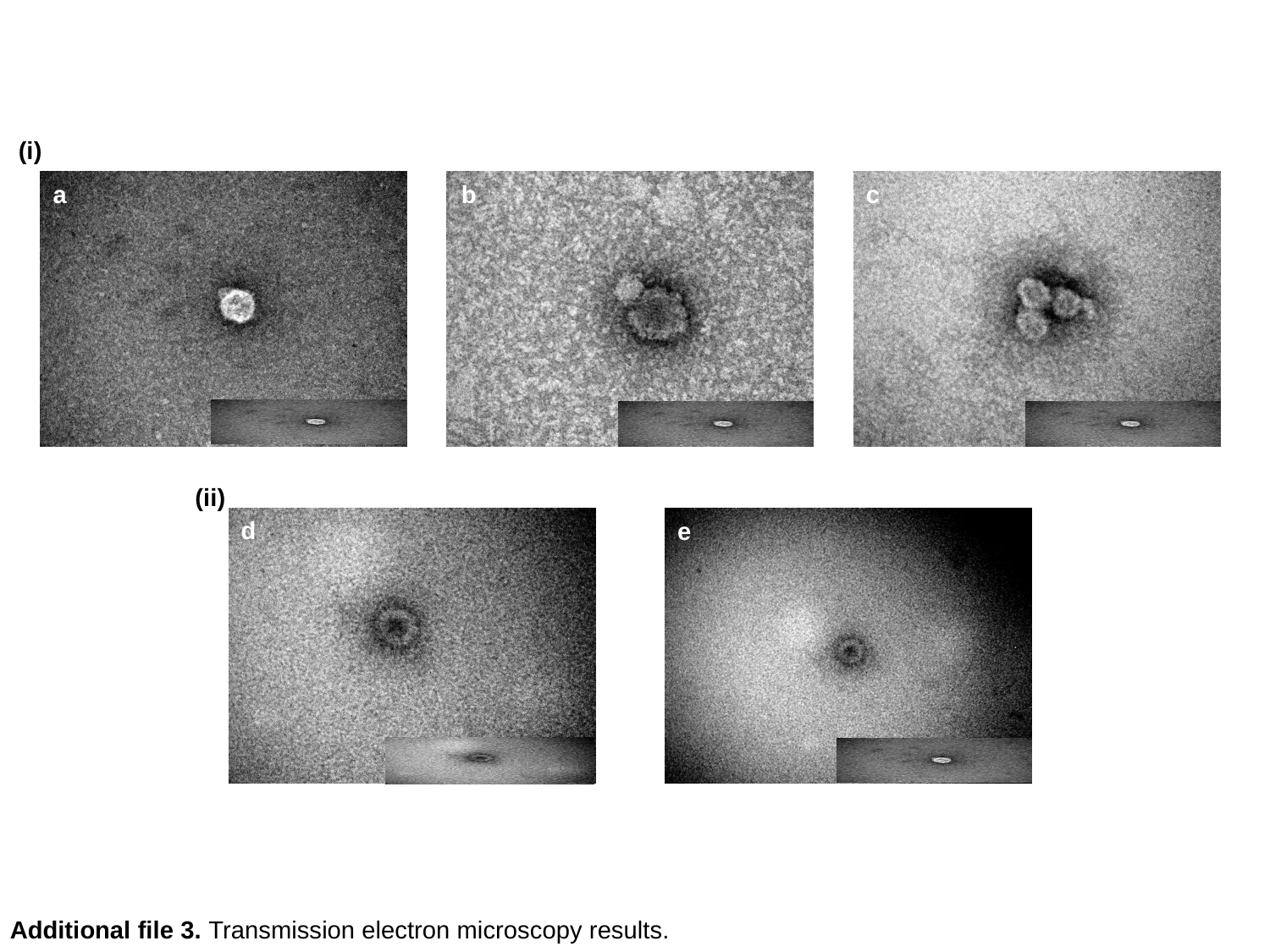

(i)
b
c
a
(ii)
d
e
Additional file 3. Transmission electron microscopy results.

Supplement: Supplementary file 1 [file viruses-13-00768-s001.zip › Supplementary_File_S3.pptx]
